# The role of stereotactic radiotherapy in addition to immunotherapy in the management of melanoma brain metastases: results of a systematic review

**DOI:** 10.1007/s11547-022-01503-7

**Published:** 2022-05-23

**Authors:** Valentina Lancellotta, Laura Del Regno, Alessandro Di Stefani, Bruno Fionda, Fabio Marazzi, Ernesto Rossi, Mario Balducci, Riccardo Pampena, Alessio Giuseppe Morganti, Monica Mangoni, Celeste Lebbe, Claus Garbe, Caterina Longo, Giovanni Schinzari, Luca Tagliaferri, Ketty Peris

**Affiliations:** 1 UOC Radioterapia Oncologica, Dipartimento di Diagnostica per Immagini, Radioterapia Oncologica ed Ematologia, Fondazione Policlinico Universitario “A. Gemelli” IRCCS, Rome, Italy; 2 Institute of Dermatology, Catholic University, Rome, Italy; 3 Fondazione Policlinico Universitario A. Gemelli, IRCCS, Rome, Italy; 4 Medical Oncology, Fondazione Policlinico Universitario Agostino Gemelli IRCCS, Rome, Italy; 5 Centro Oncologico ad Alta Tecnologia Diagnostica, Azienda Unità Sanitaria Locale-Istituto di Ricovero e Cura a Carattere Scientifico di Reggio Emilia, Department of Dermatology, University of Modena and Reggio Emilia, Modena, Italy; 6 Radiotherapy Center, Azienda Ospedaliero-Universitaria di Bologna, Bologna, Italy; 7 DIMES, Alma Mater Studiorum University of Bologna, Bologna, Italy; 8 Radiation Oncology Unit, Azienda Ospedaliera Universitaria Careggi, University of Florence, Florence, Italy; 9 Université de Paris, AP-HP Dermatology, INSERM U976, Saint Louis Hospital, Paris, France; 10 Institut Gustave Roussy and Paris-Sud-Paris-Saclay University, Villejuif (C.R.), Aix-Marseille University, Marseille (J.J.G.), and Assistance Publique-Hôpitaux de Paris Dermatology and Clinical Investigation Center, Unité 976, Université de Paris, Hôpital Saint-Louis, Paris, France

**Keywords:** Stereotactic radiotherapy, Immunotherapy, Brain metastases, Overall survival, Toxicity

## Abstract

Aim of this study was to systematically review the literature to assess efficacy and safety of stereotactic radiotherapy (SRT) in combination with immunotherapy for the treatment of melanoma brain metastases (MBM). The literature was searched using PubMed, Scopus, and Embase. Studies comparing SRT *plus* immunotherapy *versus* SRT or immunotherapy alone were deemed eligible for inclusion. Two studies showed improved overall survival after SRT *plus* immunotherapy in melanoma cancer patients with brain metastases. Three studies reported data on LC and DFS showing as SRT *plus* immunotherapy did not improve local control and DFS rates. G3-G4 toxicity was reported in only one study (20% in the SRT *plus* immunotherapy group *versus* 23% in the immunotherapy group). Despite SRT *plus* concurrent immunotherapy seems associated with possible survival advantage and low ≥ G3 late toxicity rates, the quality of evidence is very low. Therefore, in patients with brain metastases from melanoma, SRT *plus* immunotherapy should be evaluated on an individual basis after discussion by a multidisciplinary team.

**Supplementary Information:**

The online version contains supplementary material available at 10.1007/s11547-022-01503-7.

## Introduction

Immune checkpoint inhibitors and targeted therapies have demonstrated to enable long-term disease control in a high percentage of patients with metastatic melanoma; however, prognosis of patients with melanoma brain metastases (MBM) remain poor, with 17–22 weeks median overall survival from the time of diagnosis [1–3].

Data on the efficacy of immunotherapy in patients with MBM are limited having generally been excluded from clinical trials. In phase II studies, where patients with metastatic melanoma and at least one brain metastasis were included, both anti-CTLA-4 (ipilimumab) and anti-PD1 (nivolumab and pembrolizumab) antibodies achieved response rates of 10–24% for ipilimumab [4], 20–22% for nivolumab and pembrolizumab [5, 6], and 46–57% for the ipilimumab *plus* nivolumab combination. [6, 7]

At the same time, SRT became largely available and nowadays it was increasingly used in MBM. In fact, stereotactic radiotherapy (SRT) in patients with MBM plays a key role due to excellent local control rates, minimal invasiveness, and possibility to repeat the treatment in case of new lesions. The effects of radiotherapy (RT) on both tumour cells and non-malignant tissues implicate a complex interaction with the immune system, resulting in both immunostimulatory and immunosuppressive outcomes. Advantages of RT are the enhancement of the immune-checkpoint inhibitors antitumour effects through increased production of cytokines and endogenous danger signals, tumour microenvironment changes, promotion of tumour-associated antigens presentation on antigen- presenting cells, and stimulus to T cell repertoire diversification [8]. Based on these biological mechanisms, RT *plus* concurrent immunotherapy could be a combined modality treatment theoretically associated with improved patient outcome. However, synergy between RT and immunotherapy should be clinically and radiologically supervised, due to potentially increased risk of either RT-induced or immune-mediated toxicity. In particular, RT-induced toxicity could be increased by endothelial apoptosis and neuroinflammation produced by immunotherapy. The risk of brain toxicity is associated with tumour size, exposure to higher doses of RT, and concurrent use of chemotherapy. The safety of this combined approach has been extensively analysed in a recent review including data from a large number of retrospective studies [9]. The review suggests that the combination of intracranial RT and immunotherapy has an acceptable safety profile. In addition to these findings, Kroeze et al. showed that cranial SRT is well tolerated when combined with most immune or targeted therapies [10].

The present systematic review was performed to assess the efficacy and the safety of SRT in combination with immunotherapy for the treatment of MBM compared to SRT alone or immunotherapy alone in terms of overall survival, local control, disease free survival, melanoma specific survival, and late ≥ G3 toxicity.

## Materials and methods

### Development of clinical question

The clinical question was developed based on the P.I.C.O. framework as: population (P), intervention (I), comparison (C), and outcomes (O). The clinical question was: (P) in melanoma brain metastases, is SRT *plus* immunotherapy (I) superior when compared to SRT alone or immunotherapy alone (C), in relation to the outcomes (O) of benefit and harm? **Supplementary 1** reports the development of GRADE (Grades of Recommendation, Assessment, Development and Evaluation) Recommendation.

### Search strategy and selection of evidence

**Fig. 1:**
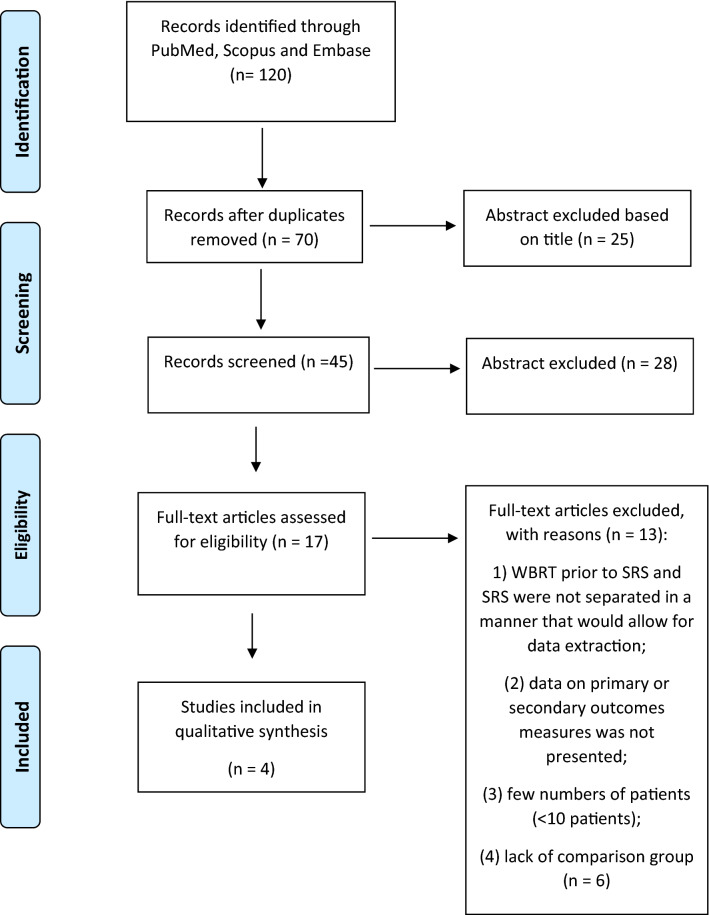
PRISMA Flow-chart for outcomes and toxicity

The systematic review was conducted in accordance with the PRISMA guidelines [11]. We performed a comprehensive literature search using PubMed, Scopus, and Embase (up to July 2020) to identify the full articles evaluating efficacy and safety of SRT *plus* immunotherapy in brain metastases from melanoma (Fig. 1). ClinicalTrials.gov was searched for ongoing or recently completed trials, and PROSPERO was searched for ongoing or recently completed systematic reviews (Supplementary 2). Electronic search was supplemented by manually searching the references of included studies and review articles. The studies were identified using the following medical subject headings (MeSH) and keywords: “melanoma”, “immunotherapy”, “radiotherapy”, “toxicity”, “brain metastases”. The search strategy was: (“melanoma” [Mesh] OR “melanoma” [All fields]) AND (“radiotherapy” [Mesh] OR “radiation therapy” [All fields]), AND “immunotherapy” [Mesh] OR “immunotherapy” [All fields]) AND “toxicity” [Mesh] OR “toxicity” [All fields] AND “neoplasm metastases” [Mesh] OR “brain metastases” [All fields]). The search was restricted to papers published in English. In order to avoid the missing of relevant studies, we chose this strategy burdened by high sensitivity and low specificity. We analysed only clinical studies presented as full texts and reporting on patients with MBM who underwent SRT *plus* immunotherapy. Conference papers, surveys, letters, editorials, book chapters, case reports, and reviews were excluded. Time restriction (2010– July 2020) of the publication was considered. Studies were identified through a search process performed by three independent reviewers (VL, LDR, BF), and uncertainty regarding eligibility was resolved by a multidisciplinary committee (ADS and RP—Dermato-oncologist expert in melanoma, MB—Radiation Oncologist expert in SRT, FB—Medical and Radiation Oncologist expert in immunotherapy, ER—Medical Oncologist expert in immunotherapy). Eligible citations were retrieved for full-text review. An external expert committee defined the outcomes of benefit and harm (CG, CeL, CaL, AGM, MM). A multidisciplinary master board (GS—Medical Oncologist expert in skin cancer, LT—Radiation Oncologist expert in skin cancer, KP—Dermato-oncologist expert in dermato-oncology) coordinated the project and performed the final independent check and the definitive approval of the review. The GRADEpro Guideline Development Tool (GDT) (McMaster University, 2015) was used to create Summary of Findings tables in Cochrane systematic reviews (Supplementary 3). The quality assessment showed high clinical and methodological heterogeneity and risks of bias in the included studies, making quantitative synthesis inappropriate. Therefore, meta-analysis outcomes were not reported.

### Identification of outcomes

The external expert committee identified the following outcomes of benefit: overall survival (OS, defined as the time from baseline to death from any cause or last follow-up), melanoma specific survival (MSS, defined as the time from baseline to death from disease cause or last follow-up), disease free survival (DFS, defined as time from baseline to clinical or radiological progression) and local control (LC, defined as time from baseline to cancer detected in the treated site at any time after initial treatment). The identified outcome of harm included acute and late ≥ G3 toxicity. All these outcomes were considered as “critical” for the decision-making process.

### Quality of evidence evaluation

Certainty of evidence for all selected outcomes was performed according to the GRADE approach, considering study limitations, imprecision, indirectness, inconsistency, and publication biases. Certainty level starts at higher pre-specified level for randomized controlled trials, but levels of certainty can be downgraded if limitations in one of the above-mentioned domains are detected. Evidence was classified as having high, moderate, low, and very low level of certainty.

### Benefit/harm balance and clinical recommendation

Based on the summary of evidence, the following judgments about the benefit-to-risk ratio between intervention and comparison were stated: favourable, uncertain/favourable, uncertain, uncertain/unfavourable, and unfavourable (both for intervention or comparison). The strength of the recommendation is considered as strong positive, conditional positive, uncertain, conditional negative, or strong negative.

## Results

### Search strategy results and details of the identified relevant studies

Seventy potentially relevant studies were identified through the database searches after duplicates and title removal. After screening of title and abstract, 53 papers were excluded. Of the remaining 17 papers, 13 were excluded through the full text examination for the following reasons: (1) the majority of patients received whole brain RT prior to SRT and those who received SRT were not separated in a way allowing for data extraction; (2) a small minority of patients received SRT but data on their primary or secondary outcomes measures was not reported and therefore could not be extracted for the analysis; (3) few patients (< 10) were enrolled; (4) a comparison group was lacking (Fig. 1). At the end of the process, four original manuscripts were selected for analysis.

### Studies characteristics

The systematic review was performed on a total of 367 patients with melanoma brain metastases included in four studies published from 2010 to 2020 [12–15]. Patient demographics, treatment characteristics, OS, LC, DFS, MSS, and toxicity data were recorded (Table 1). The results of excluded studies are presented in Supplementary Table 4. The median follow-up time ranged between 0.2 and 58.4 months (median: 6.9 months).

**Table 1:** Patient demographics, treatment characteristics, OS, LC, DFS, MSS and toxicity

Author	Period	Study	Sample size, n	Gender	Median age, years	Median number of lesions	MSS	LC	DFS	OS	Toxicity	Median FU (months)
Tommer-Nestler (12)	2011–2016	Retrospective	**SRT + I**: 13 **SRT**:13 (48 lesioni)	**SRT + I** F:13 M:6 **SRT** F:5 M:8	**SRT + I**: 54.9 **SRT:** 61.5	**SRT + I:** 1–5 **SRT:** 1–5		**SRT + I**: at 6 months 86% **SRT**: 80%			G3-G4:0%	6
Tetu (13)	2013–2017	Retrospective	**SRT + I**:81 **I**:169	**RT + I** M: 53 F: 40 **I** M: 98 F: 71	**RT + I:** 58 (50–67) **I:** 63 (50–71)				**SRT + I:** 6-m:44.4% 18-m: 23.1% **I:** 6-m:31.2% 18-m: 17.1%	**SRT + I**: 1-y: 58.9% 2-y: 37.4% **I**: 1-y: 33.8% 2-y: 22.4%	**G3-G4** **SRT + I**:20% **I:**23%	6.9 ( 0.2–58.4)
Mathew (14)	2008–2011	Retrospective	**SRT + I**: 25 **SRT**:33 198 lesions	**SRT + I** F:12 M:13 **SRT** F:16 M:17	**SRT + I:** 62 (27–87) **SRT:** 57 (27–91)	**SRT + I:** 3 (1–9) **SRT:** 3 (1–9)		**SRT + I:** 6-m: 63% **SRT:** 6-m: 65%	**SRT + I:** 6-m: 35% **SRT:** 6-m: 47%	**SRT + I:** 6-m: 56% **SRT:** 6-m: 45%	**SRT + I:** hemor: 7 **SRT:** hemor: 10	6 (0.3–47)
Silk (15)	2005–2012	Retrospective	**RT + I**: 33 (SRT 17) **RT**:37 (SRT:16)	**RT + I** M: 20 F: 13 **RT** M: 20 F: 17	**RT + I** 56.6 **RT** 57.7	1–14				**SRT + I** 19.9 m **SRT** 4 m	**SRT + I:** hemor: 1 **SRT:** hemor: 4 radionecrosis:3	

DFS, disease specific survival; FU, follow-up; G, grading; I, immunotherapy; LC, local control; m, months; MSS, melanoma specific survival; OS, overall survival; SRT, stereotactic radiotherapy; RT, radiation therapy; y, year; hemor, intracranical haemorrhage

The median age for patients treated with SRT *plus* immunotherapy was 56.6 years (range: 27–87 years) and for SRT or immunotherapy alone was 54.5 years (range: 27–91 years). The most commonly used immunotherapy drugs were pembrolizumab and nivolumab, which were used in two out of four studies [12, 13]. Ipilimumab was administered in three studies [13–15]. The most commonly used SRT average dose was 20 Gy (range: 14–24 Gy). In the included studies, SRT was administered via Gamma Knife in one study [14] and Cyberknife in another study [12], while in the other two studies the treatment machines was not specified [13, 15]. Ninety-one patients received whole brain irradiation after SRT [13, 15]. One hundred sixty-four patients presented extracranial disease at the time of brain metastases diagnosis. One hundred forty-two patients had solitary brain metastases. In the SRT *plus* immunotherapy group and in the SRT or immunotherapy group, 90 and 145 patients underwent prior systemic therapy, respectively.

### Studies description

The retrospective study of Trommer-Nestler et al [12]. compared SRT *plus* immunotherapy to SRT alone in patients with MBM. Twenty-six patients were treated either with SRT alone (13 patients; 20 lesions) or in combination with anti-PD-1 (13 patients; 28 lesions). Simultaneous treatment was defined as the somministration of the first dose of PD-1 inhibitor at least one week before SRT treatment and for at least six weeks after irradiation (n = 13). Non-simultaneous treatment (n = 13) was defined as the delivery of SRT at least three months after the last cycle of immunotherapy and at least six months before the first cycle. Patients with simultaneous treatment received at least three cycles of anti-PD-1 therapy with either pembrolizumab at a dose of 2 mg/kg every three weeks (n = 10) or nivolumab at a dose of 3 mg/kg every two weeks (n = 3). Eight (62%) of the combined treated patients were positive for BRAF mutation, five (38%) for NRAS mutation, and one for c-KIT mutation. In the SRT group, 10 (77%) patients were positive for BRAF mutation, one for NRAS mutation, and one for c-KIT mutation. A median of two (range: 1–5) lesions per patient were treated in the SRT *plus* anti-PD-1 group and a median of one (range: 1–3) in the SRT group. The median lesion size for the SRT *plus* anti-PD-1 group was 0.6 cm^3^ (range: 0.1–7.4 cm^3^) and 0.1 cm^3^ (range: 0.01–2.7 cm^3^) in the SRT group.

A prospective non-randomized study [13] compared the role of SRT *plus* immunotherapy *versus* immunotherapy alone in patients with MBM. Simultaneous treatment was defined as the delivery of radiotherapy treatment 30 days before the first systemic therapy dose to 30 days after the first dose of the same therapy line. Non-simultaneous treatment was defined as the delivery of SRT ≥ 30 days before the start of the same therapy line. No significant differences in gender, LDH, BRAF status, age, performance Karnofsky status (KPS), tumour volume, number of lesions, and incidence of extracranial disease were found between the two groups. Fifty-eight (62%) of the simultaneously treated patients and ninety-six (57%) of the immunotherapy group were positive for BRAF mutation. Thirty-nine (42%) patients in the combined group and sixty-four (38%) in the immunotherapy group had more than three lesions.

In line with this study, Mathew et al. [14] compared the role of SRT *plus* ipilimumab to SRT alone in patients with MBM, and found no significant differences in age, KPS, number of lesions, tumour volume, and incidence of extracranial disease between the two groups. A median of three (range: 1–9) lesions per patient was treated in the SRT *plus* ipilimumab group and in the SRT group. The median lesion size in the SRT *plus* ipilimumab group and in the SRT group was 0.6 cm^3^ (0.1–4.6 cm^3^) and 1.7 cm^3^ (0.2–8 cm^3^), respectively.

Finally, Silk et al. [15] retrospectively evaluated the role of SRT in combination with immunotherapy *versus* SRT alone in patients with brain metastases of melanoma. There were no significant differences between the SRT *plus* ipilimumab group and the comparison group. Seventeen (51.5%) of the simultaneously treated patients and three (25%) of the SRT group were positive for BRAF mutation. Fifteen (45.4%) patients in the combined group and twenty-one (56.7%) in the SRT group had 1–3 lesions.

### Outcomes of harm

All four studies reported data on toxicity (Table 2). G3-G4 toxicity was reported in only one study (20% in the SRT plus immunotherapy group versus 23% in the immunotherapy group). Overall, of the 136 patients treated with SRT plus immunotherapy combination12-15, G2 intracranial haemorrhage was reported in eight (5.9%) patients14,15, headache in six (4.4%; G1: 2.9%, G2: 1.5%)12, nausea in two (1.5%; G1: 0.75%, G2: 0.75%)12, vertigo in four (2.9%; G1: 2.15%, G2: 0.75%)12, and fatigue in three (2.15%)0.12. Moreover, thyroid disorders were detected in five patients (3.7%; G1: 2.2%, G2: 1.5%)12,13 and gastrointestinal G2 toxicity in two patients (2.9%)0.12 Of 231 patients who were treated with SRT or immunotherapy alone, G2 intracranial haemorrhage was reported in 11 patients (4.8%)14,15, G2 radiation necrosis in three (1.3%)15, G1 headache in three (1.3%), G1 nausea in one (0.4%), G1 vertigo in two (0.8%), fatigue in three (1.3%, G1: 0.9% and G2: 0.4%)12, and thyroid disorders in one (G1: 0.4%)0.13.

**Table 2:** Toxicitiy in SRT + IT group

Study	ICI Target	Toxicities	Steroids
Trommer-Nestler et al., 2017 [12]	PD-1	G1: (30%) G2: (15%) Headache G1: (7%) G2: (7%) Nausea G1: (23%) G2: (7%) Vertigo G1: (23%) G2: (7%) Fatigue G1-2: (30%) Thyroid disorder G1-2: (15%) Gastroenterological symptoms	NR
Tetù et al., 2019 [13]	CTLA-4 or PD-1	G3-4: Adrenal insufficiency (8%) G3-4: Transaminase increased (2%) GGT increased (2%) G3-G4: Tyroid disorder (2%) G3-G4: Dyspnea (2%)	NR
Mathew et al., 2013 [14]	CTLA-4	G: 1–2 ICH (4.8%)	Patients with symptomatic ICH received short-course steroids
Silk et al., 2013 [15]	CTLA-4	G: NR ICH (5.8%)	NR

G, grading; ICH, intracranial hemorrhage; PD-1, Programmed Death 1; CTLA-4, Cytotoxic T-Lymphocyte-Associated protein; NR, not reported; SRT, stereotactic radiotherapy; IT, immunotherapy

The certainty of evidence was considered as “very low” for each outcome of harm for the following reasons: indirectness for population including both target therapy and immunotherapy13, imprecision for sample size12-15, and finally to possible selection bias due to a sub-group analysis. [13].

### Outcomes of benefit

Three studies reported OS rates [13, 15] and two of them showed improved OS after SRT *plus* immunotherapy in melanoma cancer patients with brain metastases [13, 15]. Three studies reported data on LC and DFS showing as SRT *plus* immunotherapy did not improve local control [12–14] and DFS rates [13, 14]. Finally, no studies reported data about MSS. The Summary of Findings table for outcomes of benefit is reported in Table 3. The certainty of evidence was judged as “very low” for each outcome of benefit for the following reasons: indirectness for population including both target therapy and immunotherapy [13], imprecision for sample size [12–15], and finally to possible selection bias due to a sub-group analysis. [13]

**Table 3:** Summary of Findings table for outcomes of benefit

Certainty assessment							No of patients		Effect		Certainty	
No of studies	Study design	Risk of bias	Inconsistency	Indirectness	Imprecision	Other considerations	SRT + IT	SRT or IT alone	Relative(95% CI)	Absolute(95% CI)		
**OS (Mathew, Melanoma Research 2013) (follow up: median 6 months; evaluated with: event)**												
1	Observational	Serious ^a^	Not serious	Not serious	Serious ^b^		SRT + Ipilimumab 6-month OS 56% vs SRT alone 45% (P = 0.18))				- Very low	
**OS (Silk, Cancer Medicine 2013) (follow up: mediana 10 mesi; valutato con: eventi)**												
2	Observational	Serious ^c^	Not serious	Not serious	Serious ^b^		SRT + Ipilimumab 19.9 months vs SRT alone 4 months (HR: 0.31; P = 0.009)				- Very low	
**LC (Trommer-Nestler, International Journal of Molecular Sciences 2018) (follow up: median 6 months; evaluated with: event)**												
1	Observational	Serious ^a^	Not serious	Not serious	Serious ^b^		6-months LC 86% in the SRT + IT group vs. 80% in the SRT alone group (p = 0.028)				- Very low	
**LC (Mathew, Melanoma Research 2013) (follow up: median 6 months; evaluated with: event)**												
2	Observational	Serious ^a^	Not serious	Not serious	Serious ^b^		6-months LC 63% SRT + IT group and 65% SRT alone group (p = 0.55)				- Very low	
**DFS (Mathew, Melanoma Research 2013) (follow up: median 6 months; evaluated with: event)**												
1	Observational	Serious ^a^	Not serious	Not serious	Serious ^b^		6-months DFS 35% SRT + IT group and 47% SRT alone group (p = 0.48)				- Very low	
**OS (Tetù, European Journal of Cancer 2019) (follow up: median 6.9 months; evaluated with: event)**												
1	Observational	Serious ^b^	Not serious	Serious ^c^	Not serious		1-year and 2-year OS were, respectively, 58.9% (95% CI:49.2–70.5) and 37.4% (95% CI: 27.6–50.7) for the SRT + IT group and 33.8% (95% CI: 27–42.5) and 22.4% (95% CI: 16.1–31.3) for the IT group (HR Z 0.60, 95% CI 0.4 to 0.8; p = 0.007)				- Very low	
**DFS (Tetù, European Journal of Cancer 2019) (follow up: median 6.9 months; evaluated with: event)**												
1	Observational	Serious ^d^	Not serious	Serious ^c^	Not serious		The 6-month and 18-month PFS was 44.4% (95% CI: 35.1–56.2) and 23.1% (95% CI: 15.5–34.4) in the cRT group and 31.2% (95% CI: 24.8–39.4) and 17.1% (95% CI: 11.8–24.7) in the no-cRT group, respectively (p = 0.23)				- Very low	
**LC (Tetù, European Journal of Cancer 2019) (follow up: median 6.9 months; evaluated with: event)**												
1	Observational	Serious ^d^	Not serious	Serious ^c^	Not serious		disease control rate were similar between the two groups (37% and 59% in the cRT-group versus 31% and 60% in the no-cRT group, respectively; p = 0.8)				- Very low	

CI, Confidence interval; SRT, stereotactic radiotherapy; IT, immunotherapy; OS, overall survival; DFS, disease-free-survival; LC, local control; RT, radiotherapy; HR, hazard ratio

^a^Selection bias

^b^Small population

^c^Possible selection bias due to a sub-group analysis

^d^Included immunotherapy and target therapy

### Evidence to decision framework

In the MBM setting, the proposed intervention (SRT *plus* immunotherapy) did not increase the incidence of side effects (treatment-associated brain toxicity) compared to the control one (SRT or immunotherapy alone). Moreover, SRT *plus* immunotherapy has proven effective in improving OS without benefit in terms of LC and DFS.

### Benefit/harm balance and final recommendation

The panel voted for the benefit/harm as uncertain. The strength of the recommendation was voted as conditionally weak by all five panel members. Hence, the final recommendation of the panel was: “In patients with melanoma brain metastases, the combination of SRT *plus* immunotherapy should be evaluated on an individual basis through discussion by a multidisciplinary team”.

## Discussion

The modern approach to cancer patients is based on personalized treatment. Integration between different therapies, especially if loco-regional and systemic, can offer remarkable clinical benefit to patient in terms of oncological outcomes and particularly of OS.

In our systematic review, we examined whether SRT *plus* immunotherapy is superior to SRT alone in terms of benefit and harm balance in patients with MBM. The expert panel suggested the use of SRT *plus* immunotherapy in patients with MBM after discussion about each patient by the multidisciplinary team. This underlines the lack of data regarding the effect of timing and type of immunotherapy on the outcome after SRT of MBM. The results of the present systematic review suggest that (1) immunotherapy and SRT have an almost additive effect considering the improved OS recorded in the combined modality treatment group, and (2) local response is greater and faster after SRT *plus* concurrent immunotherapy compared to SRT or immunotherapy alone. Heterogeneity in treatment protocols (SRT total dose, schedule, isodose prescription) and in the definition of "simultaneous" SRT *plus* immunotherapy combination, small patients population included in the selected studies, and selection biases could have affected the possibility to accurately define the effective role of SRT *plus* immunotherapy in this setting [12–15]. Therefore, we reported only a qualitative literature analysis.

Despite this limitations, two studies showed that the combination of SRT *plus* immunotherapy in MBM patients improves OS [13–15] without significant improvement of LC [12–14] and DFS rates [13, 14]. Moreover, one study showed a significant improvement of OS also in patients receiving immunotherapy after SRT [15]. This finding is supported by a small number of retrospective clinical studies (not included in the present review) evaluating the timing effect in the combination of SRT and immunotherapy for MBM [16–19]. Kiess et al. reported improved OS in patients treated with SRT before or during ipilimumab compared to those treated with SRT after ipilimumab [16]. Similarly, Schoenfeld et al. reported improved OS after SRT delivered before ipilimumab compared to SRT after ipilimumab. [17].

However, even if confirmed by the latter studies, the results of our analysis present paradoxical aspects. Indeed, an improvement in OS without prolonged LC and DFS is hard to be explained. Since this review is based on retrospective studies, selection bias could be hypothesized and in particular a preferential referral of patients with better performance status or with less relevant comorbidities to the combined modality treatment [18].

Nevertheless, these results are similar to the ones recorded in other oncological settings, showing an increased effect of immunotherapy given after RT. For example, the PACIFIC trial evaluated activity and safety of durvalumab administered after combined chemoradiation in patients affected by stage III NSCLC. In that trial, median time to death or distant metastasis was longer with durvalumab compared to placebo (23.2 months *vs* 14.6 months; p < 0.001) [19]. Radiotherapy may be a synergistic effect with immunotherapy. Indeed, RT can cause a transient alteration in the blood–brain barrier [20], resulting in an uptake of immunotherapy. Moreover, the combination of RT and immunotherapy may increase systemic antitumour response [20] and it could lead to an abscopal effect, correlating with prolonged survival [21].

In any case, if the positive effect of the SRT *plus* immunotherapy combination was confirmed, it would be useful to define which type of immune checkpoint inhibitor is more effective when combined to SRT. Lehrer et al. [22]. suggested that the association of anti-PD-1 and SRT results in a greater and faster tumour shrinkage compared to anti-CTLA-4 *plus* SRT. However, these results may have been influenced by a higher number of lesions in the anti-PD-1 *plus* SRT group [23].

In the present systematic review, SRT *plus* immunotherapy combination was found to have tolerable toxicity profile. In the SRT *plus* immunotherapy group (136 patients) intracranial haemorrhage was recorded only in eight patients [14, 15], headache in six, nausea in two, vertigo in four, and fatigue in four [12]. In the SRT or immunotherapy alone group (231 patients), intracranial haemorrhage was recorded in eleven [14, 15], radiation necrosis in three patients [15] headache in three, nausea in one, vertigo in two, and fatigue in three [12]. In patients included in this analysis, adverse events severity was never scored as higher than Grade 2 according to the CTCAE scale.

Limitations of our systematic review include the small number of studies, especially for the secondary outcome measures. In addition, none of them was a randomized controlled trial and all studies were retrospective. Toxicity data were extrapolated from retrospective studies with different adverse effects categorization and in some cases with short follow-up. More generally, a median follow-up of 6.9 months (range: 0.2–58.4 months) is insufficient to allow a proper assessment of late toxicity. However, longer follow-up is unlikely to lead to the detection of further differences in late adverse events between patients treated with SRT *plus* immunotherapy compared to single treatment groups.

## Conclusions

The final recommendation released by the panel was: “In patients with brain metastases from melanoma, SRT *plus* immunotherapy should be evaluated on individual basis after discussion by a multidisciplinary team”. The communication with the patient should include the following topics: prognosis with and without treatment, limited power of evidence on the benefit derived from treatment combination, and treatment related risks including haemorrhage and radiation necrosis.

## Supplementary Information

Below is the link to the electronic supplementary material.

Supplementary file1 (DOCX 13 kb)

Supplementary file2 (DOCX 19 kb)

Supplementary file3 (DOCX 19 kb)

Supplementary file4 (DOCX 13 kb)
