# Supplementary material for: The role of stereotactic radiotherapy in addition to immunotherapy in the management of melanoma brain metastases: results of a systematic review
Source: Radiol Med. 2022 May 23;127(7):773–83. doi: 10.1007/s11547-022-01503-7 (PMC9308608; doi:10.1007/s11547-022-01503-7)
Supplement: Supplementary file 1 — Supplementary file1 (DOCX 13 kb) [file 11547_2022_1503_MOESM1_ESM.docx]

# Supplementary Table 1: Population, Intervention, Control, Outcome

| **Population, Intervention, Control, Outcome, Study Design (PICO) criteria.** | |
| --- | --- |
| **Population** | Patients with melanoma brain metastases |
| **Intervention** | Stereotactic radiotherapy and immunotherapy |
| **Control** | Stereotactic radiotherapy alone or immunotherapy alone |
| **Outcomes** | OS, LC, MSS, DFS, incidence of late ≥ G3 toxicity |

Abbreviations: LC: local control; OS: overall survival; MSS: melanoma specific survival; DFS: disease-free survival; G: grading
