# Supplementary material for: The role of stereotactic radiotherapy in addition to immunotherapy in the management of melanoma brain metastases: results of a systematic review
Source: Radiol Med. 2022 May 23;127(7):773–83. doi: 10.1007/s11547-022-01503-7 (PMC9308608; doi:10.1007/s11547-022-01503-7)
Supplement: Supplementary file 2 — Supplementary file2 (DOCX 19 kb) [file 11547_2022_1503_MOESM2_ESM.docx]

**Supplementary Table 2: Ongoing clinical trials investigating immune checkpoint inhibitors and radiosurgery for brain metastases (www.** **ClinicalTrials.gov update at March 25, 2021)**

| **Study** | **Phase** | **Institution/Location** | **Histology** | **SRS dose** | **Immunotherapy** | **Description** | **Primary Outcome(s)** |
| --- | --- | --- | --- | --- | --- | --- | --- |
| NCT02886585; Recruiting | 2 | Dana Farber Cancer Institute, Boston, MA, USA | MBM | NR | Pembrolizumab | Patients with 1-4 MBM; SRS between Pembro cycles 1-2 | ORR; OS; EORR |
| NCT02858869; Recruiting | 1 | Emory University/Winship Cancer Institute, Atlanta, GA, USA | MBM  NSCLC | 30 Gy in 5 Fx  27 Gy in 3 Fx  18-21 Gy in 1 Fx | Pembrolizumab | Pembro courses q3W x 2 years; SRS between days 2-15 of course 1 | DLT |
| NCT03340129  (ABC-X Study);  Recruiting | 2 | Melanoma Institute Australia, Sydney, New South Wales, Australia | MBM | 16-22 Gy in 1 Fx | Nivolumab + Ipilimumab | Induction Nivo + Ipi; if disease progression on ICI then SRS | Intracranial response to ICI |
| NCT02716948; Recruiting | 1 | Sidney Kimmel Comprehensive Cancer Center/Johns Hopkins University, Baltimore, MD, USA | MBM | NR | Nivolumab | Nivo q2W; SRS 8 days after 1^st^ dose of Nivo | Incidence of SAE |
| NCT02097732; Active, not recruiting | 2 | University of Michigan Cancer Center, Ann Arbor, MI, USA | MBM | NR | Ipilimumab | SRS with Ipi 2-3 W after q3W vs. Ipi x 2 q3W then SRS then Ipi x 4 | 6-month LC |
| NCT01703507;  Active, not recruiting | 1 | Sidney Kimmel Cancer Center at Thomas Jefferson University, Philadelphia, PA, USA | MBM | NR | Ipilimumab | Ipi q3W with SRS on day 1 in week 1 | MTD |
| NCT03297463;  Not yet recruiting | 1/2 | Masonic Cancer Center, University of Minnesota, Minneapolis, MN, USA | MBM | NR | Ipilimumab + IL-2 | SRS or WBRT followed by IL-2 + Ipi | MTD |

Abbreviations: DLTs: dose-limiting toxicities; EORR: extracranial overall response rate; Gy: gray; Fx: fraction; IL: interleukin; Ipi: ipilimumab; LC: local control; MBM: melanoma brain metastases; MTD: maximum tolerated dose; Nivo: nivolumab; NR: not reported; ORR: overall response rate; OS: overall survival; Pembro: pembrolizumab; PFS: progression-free survival; q: every; RCC: renal cell carcinoma; SAE: severe adverse effects; SRS: stereotactic radiosurgery; W: weeks; WBRT: whole brain radiation therapy
