# Supplementary material for: The role of stereotactic radiotherapy in addition to immunotherapy in the management of melanoma brain metastases: results of a systematic review
Source: Radiol Med. 2022 May 23;127(7):773–83. doi: 10.1007/s11547-022-01503-7 (PMC9308608; doi:10.1007/s11547-022-01503-7)
Supplement: Supplementary file 4 — Supplementary file4 (DOCX 13 kb) [file 11547_2022_1503_MOESM4_ESM.docx]

**Supplementary Table 4. Excluded papers**

| **Author** | **Journal** | **Study** | **Exclusion reason** |
| --- | --- | --- | --- |
| K. A. Ahmed | Annals of Oncology 27: 2288–2294, 2016 | Retrospective | Patients treated with SRT and various systemic immunologic and targeted melanoma agents; lack of comparison group |
| Yi An | Radiother and Oncol 2017; 125 80–88 | Retrospective | Lack of comparison group |
| Patricia D. Banks | Health Sci Rep. 2019;2:11 | Retrospective | Stereotactic radiosurgery and/or whole brain radiotherapy; the patients who received SRT were not separated in a manner that would allow for data extraction; lack of comparison group |
| J G. D. Cannon | JAAD Case Reports 2018; 4:248-50 | Retrospective | Few numbers of patients (<10); lack of comparison group |
| R Carron | European Journal of Cancer 135 (2020) 52-61 | Retrospective | Lack of comparison group |
| E S Choong | European Journal of Cancer 2017; 75:169-178 | Retrospective | WBRT was used in addition to SRS in 42 (38.9%) patients; the patients who received SRT were not separated in a manner that would allow for data extraction; various systemic immunologic and targeted melanoma agents |
| Or Cohen-Inbar, | J Neurosurg 127:1007–1014, 2017 | Retrospective | Stereotactic radiosurgery and/or whole brain radiotherapy; the patients who received SRT were not separated in a manner that would allow for data extraction; data on primary or secondary outcomes has not be extracted for the analysis |
| P Fang | J Neurooncol 2017; 133:595–602 | Retrospective | Lack of comparison group |
| P Gabani | Radiother and Oncol 2018; 128: 266–273 | Retrospective | WBRT: 741 (67.1%) |
| B Gatterbauer | Cancer Medicine. 2020;9:4026–4036 | Retrospective | Patients treated with various systemic immunologic and targeted melanoma agents |
| C. Gaudy-Marqueste | European Journal of Cancer 2017; 84: 44-54 | Retrospective | Patients treated with various systemic immunologic and targeted melanoma agents |
| A P Kiess | Int J Radiat Oncol Biol Phys. 2015; 92(2): 368–375 | Retrospective | Lack of comparison group |
| F Martins | Journal of Neuro-Oncology 2020; 146:181–193 | Retrospective | Patients treated with various systemic immunologic and targeted melanoma agents: lack of comparison group |

Abbreviations: SRT: stereotactic radiotherapy; WBRT: whole brain radiotherapy
